# Pestilential Streams

**Published:** 1920-08-14

**Authors:** 


### Pestilential Streams.

### PROTESTS BY DURHAM M.O.H.

In a report to the County Council the Medical Officer of Health for Durham states that, considering the present unsatisfactory state of many rivers and streams, and also the many representations that have been made to offenders with little success, the attention of the Ministry of Health might with advantage be called to the urgent need for further legislation to give effect to the recommendations of the Royal Commission on Sewage Disposal. The Medical Officer mentions that in the Durham urban district the banks of the Wear from Sands House to below Kepier Hospital are lined with scavengers' refuse and present a disgusting appearance. The excuse hitherto given for these conditions is that this refuse is there to replace and repair the bank sides, which have been damaged by floods. For this purpose it is absolutely valueless without the addition of more permanent works, as in its turn it is also carried away, and much too often replaced by more refuse.

Dealing with the nuisance caused by filling up a pond with refuse by a rural district council, the Medical Officer says that when it is remembered that this particular sanitary authority (Chester-le-Street) has more power than the County Council to prevent pollution of streams within the district, it seems strange that it should advocate that the filling of this unenclosed pond with scavengers' refuse should be continued. Yet it is only in keeping with what the local authority has been doing for several years. Consequently, it will be readily understood how difficult it is to make progress when there is not only a want of co-operation, but active contravention of the statutes.

